# QTL analyses for tolerance to abiotic stresses in a common bean (*Phaseolus vulgaris* L.) population

**DOI:** 10.1371/journal.pone.0202342

**Published:** 2018-08-29

**Authors:** Lucy Milena Diaz, Jaumer Ricaurte, Eduardo Tovar, Cesar Cajiao, Henry Terán, Miguel Grajales, Jose Polanía, Idupulapati Rao, Stephen Beebe, Bodo Raatz

**Affiliations:** 1 Biotechnology Unit and Bean Program, International Center for Tropical Agriculture (CIAT), Cali, Valle, Colombia; 2 Instituto de Investigación de Recursos Biológicos Alexander von Humboldt, Bogotá, Colombia; 3 DuPont Pioneer, Salinas, Puerto Rico, United States of America; Università Politecnica delle Marche, ITALY

## Abstract

Common bean productivity is reduced by several abiotic stress factors like drought and low soil fertility, leading to yield losses particularly in low input smallholder farming systems in the tropics.

To understand the genetics of stress tolerance, and to improve adaptation of common bean to adverse environments, the BAT 881 x G21212 population of 95 recombinant inbred lines (RILs) was evaluated under different abiotic stress conditions in 15 trials across four locations in Colombia, representing two higher altitude (Darién, Popayán) and two lower altitude (Palmira, Quilichao) locations. Stress vs non-stress treatments showed that yields were reduced in drought trials in Palmira by 13 and 31%, respectively, and observed yield reductions in low phosphorus stress were 39% in Quilichao, 16% in Popayán, and 71% in Darién, respectively. Yield components and biomass traits were also reduced. Traits linked to dry matter redistribution from stems, leaves and pods to seed, such as pod harvest index and total non-structural carbohydrates, were found to be important factors contributing to yield in all conditions. In contrast, early maturity was correlated with improved yield only in lower altitude locations, whereas in higher altitudes delayed maturity promoted yield. Superior RILs that combine stress tolerance and high cross-location productivity were identified. Lines that showed good yield under strong stress conditions also performed well under non-stress conditions, indicating that breeder’s selection can be applied for both conditions at the same time.

Quantitative trait loci (QTL) analyses revealed a stable yield QTL on chromosome Pv04, detected individually in all locations, several stress treatments and in best linear unbiased predictions (BLUPs) across all trials. Furthermore, two QTL hotspots for maturity traits were identified on Pv01 and Pv08, which are the most stable QTL. The constitutive yield QTL could serve as a good candidate for marker development and could be used in marker assisted selection.

Increased understanding of the physiology of abiotic stress tolerance, combined with the availability of superior germplasm and molecular tools, will aid breeding efforts for further improvement of these plant traits.

## Introduction

Common bean (*Phaseolus vulgaris* L.) is the most important food legume for direct human consumption in the tropics of Latin America and southern and eastern Africa [1] with a total area harvested of 30.6 million ha [2]. It is an important source of protein, carbohydrates and micro minerals, particularly for smallholder farmers [2]. It is an ideal crop for the smallholder farming systems due to its symbiotic nitrogen fixing ability and relatively short growth cycle of about three months.

Bean production is affected by several constraints, mainly abiotic stresses and diseases. Hence, national averages of bush bean yields throughout the tropics typically range from 500 to 700 kg ha^-1^, significantly below the yield potential [3]. The principal abiotic stresses that limit common bean grain yield are drought and low soil fertility such as low phosphorus (P) availability and aluminum (Al) toxicity in soil. These problems tend to occur in combination [4].

Drought is estimated to affect 60% of bean production in the tropics, especially in regions such as Central America and Southern Africa [5,6] and approximately 67% of globally cultivated lands are affected by P deficits [5,7]. It has been estimated that 50% of bean production area worldwide suffers moderate to severe P limitation [8]. Low soil P availability is exacerbated in strongly acidic or alkaline soils, mainly due to formation of phosphate complexes with Al and Fe in acidic soils and Ca complexes in alkaline soils [4]. Soil acidity with pH ≤ 5.5 affect more than 50% of the word’s potentially arable land [9], and up to 60% of the acid soils in the world occur in developing countries in South America, Central Africa and Southeast Asia [10].

Conventional breeding and the selection of superior genotypes from segregating populations has been the traditional and predominant strategy in breeding programs to improve tolerance of common bean for different abiotic stress factors. Pronounced efforts were undertaken to improve drought resistance (avoidance and tolerance). Considering the complexity of this trait, indirect selection traits have been evaluated. Phenotypic differences in drought resistance were found to be associated with biomass production at mid-pod filling [11], vigorous root system [12,13], effective use of water [14], efficient dry matter redistribution to grain [14–16], phenological plasticity [17] and earliness [18,19]. Early maturity was shown to correlate with yield under stress following the strategy of drought avoidance in several studies (e.g. [13]), however, early maturity also has disadvantages including limited yield potential. Each day of reduction in growth cycle could result in a loss of 74 kg ha^-1^ [20]. This may vary depending on genotypes and conditions though, as positive as well as negative correlations of yield and maturity have been reported. The phenotypic expression of the characteristics as days to flowering (DF), days to physiological maturity (DPM) and pod harvest index (PHI) could be more influenced by the genetic constitution than by environmental stress [16].

A number of studies aimed at identifying beans adapted to low P and acid soils [5]. Several physiological responses to low P availability have been reported, such as modification of root architecture, association with mycorrhizal fungi in the root system, and higher use efficiency of absorbed P to produce biomass and grain yield [12,21–23].

Several years of field evaluation for drought resistance and acid soil tolerance identified the landrace G21212 to be adapted to these stresses. In contrast the breeding line BAT 881 is sensitive to both stresses [24–26]. Both lines belong to race Mesoamerica. The authors concluded that productivity under low P is heritable but is based on multiple mechanisms, hence, there may be opportunities to recombine genes and mechanisms and obtain higher levels of stress tolerance.

In recent years new genotyping technologies have been developed, utilizing several thousands of Single Nucleotide Polymorphisms (SNPs) that have become available through next generation sequencing (NGS). SNP based markers have become the preferred choice for genetic studies, due to their abundance and uniform distribution throughout genomes [27]. They have been applied to study diversity, genome mapping and QTLs detection for biotic and abiotic stresses in common bean [28–30]. However, adoption into breeding processes has been more limited to biotic stress resistance. Kompetitive Allele Specific PCR (KASP) genotyping technology offers high precision and robustness in assays at low cost [31]. Approximately ~1500 KASP markers have been established for the common bean community by the Generation Challenge Program, to support genetic studies and breeding programs.

The main objectives of the current study were to increase the understanding of adaptation of common bean to different stress environments, and to perform multilocational evaluations of yield stability and stress tolerance. The Mesoamerican RIL population BAT 881 x G21212 (B x G) was evaluated for yield and yield components, plant vigor, dry matter redistribution and physiological traits under different conditions of abiotic stress (drought conditions, low soil fertility and medium-high aluminum levels). The objective of this study was to identify QTLs associated with agronomic traits and to provide markers for use in marker assisted selection (MAS) employing a saturated genetic B x G map.

## Materials and methods

### Plant materials

A total of 100 common bean (*P*. *vulgaris*) genotypes were used in this study. These included 95 recombinant inbred lines (RILs) [32], the two parents (BAT 881 and G21212) used as controls and three other lines used in the bean breeding program of the International Center for Tropical Agriculture (CIAT) as additional controls in each trial. Darién, Popayán 1999 and CIAT-Palmira trials used G 3513, G 4017 (tolerant to low fertility) and DOR 364 (sensitive to low fertility) as controls; Quilichao trials used DOR 364, BAT 477 and VAX 1 (tolerant to aluminum and common bacterial blight). RILs were developed by single seed descent (SSD) from the F2 to the F5 generation, followed by seed advance to the F7 generation. BAT 881 is an advanced breeding line of CIAT, with indeterminate bush growth habit (IIa) and small light brown seed that is resistant to bean common mosaic virus (BCMV) and intermediately resistant to the leafhopper (*Empoasca kraemeri*) and angular leaf spot [32]. G21212 is a small black seeded Colombian landrace, growth habit IIIb, tolerant to low soil fertility, drought and with excellent yield, but is susceptible to BCMV [5,24,32,33]. Both genotypes showed intermediate levels of resistance to the melon thrips [32] and belong to the same race Mesoamerica of the Mesoamerican genepool.

### Description of experimental locations

Phenotypic data were obtained in four different locations in Colombia under field conditions with 10 x 10 balanced lattice design with three replicates, except three trials at Quilichao (Q02a_MAl-LP, Q03a_HAL_MP, Q03b_HAL_MP) that had two replicates. A trial refers to an evaluation of all 100 genotypes in a specific location, season, and management, a replicate represents 100 experimental plots harboring all genotypes and a block refers to a sub section of 10 plots within a replicate.

Description of locations: Darién (Dar, Inceptisol, 3° 55’N latitude, 76° 28’W longitude, 1457 masl, low P stress), Popayán (Pop, Inceptisol, 2° 25'N latitude, 76° 40'W longitude, 1730 masl, low P stress), Palmira (Pal, Mollisol, 3° 30'N latitude, 76° 21'W longitude, 965 masl, drought stress) and Santander de Quilichao (Qui, Oxisol, 3° 06’N latitude, 76° 31’W longitude, 990 masl, high Al stress). Specific soil conditions are shown in S1 Table.

Two trials were established in Darién: 1997 (September-December) with high P supply (20 kg ha^-1^ supplied, Dar97_HP) and in 1998 (September-December) with low and high P supply (9 and 90 kg ha^-1^, Dar98_LP and Dar98_HP respectively). Experimental units (4 rows of 4 m length with row-to-row distance of 0.6 m) were sown manually with 10 plants per m row length. Two trials were sown in Popayán: 1999 (April-September) with low and high P (0 and 40 kg ha^-1^, Pop99_LP and Pop99_HP,) and 2005 (June to August) with high P supply (39 kg ha^-1^, Pop05_HP). Experimental units (4 rows of 4.9 m length with row-to-row distance of 0.6 m) were tractor sown with 14 seeds per m. Two trials were established in Palmira: 2000 and 2002 in the same semester (June to September), with two levels of water supply (drought and irrigated, Pal00_D, Pal00_I, Pal02_D, and Pal02_I). Both environments were irrigated to ensure good plant establishment at early growth as needed until early pod filling. Thereafter, irrigation was discontinued in the drought treatment while irrigated or non-stress (NS) plots continued to receive supplemental irrigation of 20 mm of water twice a week for a total of additional 188 and 211 mm of water in 2000 and 2002, respectively (S1 Fig). Experimental units (4 rows of 5 m length with row-to-row distance of 0.6 m) were tractor sown with 14 seeds per m row length. Finally, four trials were sown in Santander de Quilichao: 2002 (April to June) in moderate Al saturation (40 and 64% in top 0–10 and 10–20 cm, respectively), low available P content (3.3 and 1.9 μg g^-1^) and high organic matter content (10.7 and 9.1%), supplementing with P 15 kg ha^-1^ (Qui02a_MAl_LP). A second trial in 2002 (October to January 2004), in moderate Al saturation (40 and 45%), moderate available P content (17.1 and 6.9 μg g^-1^) and moderate organic matter content (5.6 and 5.2%) soil with low P supply (10 kg ha^-1^, Qui02b_MAl_MP). In 2003 (March to June), with high Al saturations (61 and 62%), moderate available P content (23.5 and 13.1 μg g^-1^) and moderate organic matter content (5.9 and 5.4%) with low P supply (10 kg ha^-1^, Qui03_HAl_MP). Lastly, in 2003 (October to January 2004) two trials were sown with high Al (61–61% and 64–67%), high and moderate available soil P content (20.3–17.5 μg g^-1^ and 13.2–5.5 μg g^-1^), moderate organic matter content (5.6–5.4% and 6.1–5.4%), with low P supply (10 kg ha^-1^, Qui03_HAl_HP and Qui03_HAl_MP, respectively). Experimental units (2 rows of 3.72 m length in Qui02a_MAl_LP, 4 rows of 4.9 m in Qui02b_MAl_MP and 3 rows of 4.9 m length in the other Quilichao trials) were tractor sown with 14 seeds per m row length and rows separated with 0.6 m.

In the supplemental material S1 Fig the conditions of precipitation and evaporation, minimum and maximum temperature, and total rainfall where available are detailed. In all locations recommended agronomic procedures were followed.

### Phenotypic traits

Five groups of plant traits were evaluated in this study: yield components, plant vigor, dry matter redistribution, phenological traits, and mineral nutrients. Not all variables were measured in every trial.

**Yield components**: Yield (Yd, kg ha^-1^) and 100 seed weight (100SdW, g 100 seeds^-1^). At the time of harvest, 0.3 m^2^ per plot were harvested to measure seed number per area (SNA, seeds m^-2^), pod number per area (PNA, pods m^-2^). **Plant vigor**: Canopy biomass (CB, kg ha^-1^), leaf area index (LAI, m^2^ m^-2^), and leaf biomass (LB), pod biomass (PBMP) and stem biomass (SBMP, kg ha^-1^) were evaluated at mid-pod filling. Plants from 0.3 m^2^ per plot were harvested, leaves were separated from plants to measure leaf area (LI 3000; LI-CORm Inc., Lincoln, NE). Separated plant parts were oven-dried at 60°C for two days until establishing constant weight, to determine dry weight. **Dry matter redistribution**: Indices such as harvest index (HI), pod partitioning index (PPI), pod harvest index (PHI) were calculated. Seed and shoot total nonstructural carbohydrates content (TNC mg g^-1^) were measured to explore genotypic differences in dry matter redistribution and grain filling [14,16,18,33,34]. These dry matter redistribution traits were determined as HI (%): (seed biomass as dry weight at harvest) / (total canopy biomass as dry weight at mid-pod filling) x 100; PHI (%): (seed biomass as dry weight at harvest) / (pod biomass as dry weight at harvest) x 100; PPI (%): (pod biomass as dry weight at harvest) / (total canopy biomass as dry weight at mid pod filling) x 100; seed and shoot TNC were determined by comparison with glucose standards according to the method described by [35]. Because of leaf fall in common bean during seed filling, dry weight of total canopy biomass was determined at mid-pod filling [18]. Use of dry weight of canopy biomass and seed biomass at harvest time overestimates HI and PPI values in common bean. **Phenological traits**: Days to flowering (DF, days); days to harvest (DH, days). DF is defined as the number of days after planting until 50% of the plants have at least one open flower and DH is the number of days after planting until harvest. **Mineral nutrients**: One plant per plot was harvested at mid pod filling, washed with deionized water, oven-dried at 60°C for two days and ground in a 1 mm sieve. For seed evaluations seeds from 0.3 m^2^ plot area were oven-dried at 60°C for two days to be ground in a 1 mm sieve. Nutrient (N, P) composition of plant parts was determined as described by [36]. Shoot nutrient uptake was calculated as the product of shoot dry matter yield and nutrient concentration. Nutrient use efficiency (NUE_Sh, PUE_Sh) were estimated as grams of shoot biomass produced per gram of shoot nutrient uptake [37] and nutrient use efficiency for seed yield (NUE_Sd, PUE_Sd) were estimated as grams of seed biomass produced at harvest per gram of shoot nutrient uptake at mid pod filling. Additional information on description of phenotypic traits can be found in "Trait Dictionaries for Fieldbook Development" at http://mbp.generationcp.org and http://www.cropontology-curationtool.org/.

The raw data is available via dataverse https://doi.org/10.7910/DVN/ODYAPF.

### Statistical analysis

Variance and covariance analyses were carried out, and the results of the covariance are presented only for those traits that showed significant co-variation between genotypes and treatments. Pearson’s correlations between all traits were determined for all trials using data of individual replications. Data shown in this work are adjusted means for each genotype and the environment, that were obtained using SAS (v 9.3) PROC MIXED and PROC CORR [38], considering the effects of replication and blocks within replications as random and genotypes as fixed. Also least significant difference (LSD) was calculated with the same software to compare trait means. Variability and frequency distributions were represented as violin plots for each environment evaluated, generated by the statistics software R [39] with the package ggplot2 [40]. To estimate the overall adjusted means and adjusted means for each of the four locations, Best Linear Unbiased Predictions (BLUP) were used, according to Resende [41] for the four major traits (yield, 100SdW, DF, DH). We used the package lme4 [42] for analysis in the statistical software R. The following mixed linear model was defined: Y = B0 + B1 trl + B2 rep + B3 blk + B4 + err where Y is the vector of observed values, B0 is intersect, B1 is the effect of each trial, B2 the effect of each repetition, B3 the effect of each block within trials and replicates, B4 the effect of each genotype or line and err the residual error. Treatments and replicates were considered fixed effects while blocks and genotypes were considered random. The new BLUP variables were named after their respective traits (yield, 100SdW, DF, DH) across each location, e.g. Yd_D, Yd_P, Yd_C and Yd_Q, and for overall adjusted means Yd_all. We also calculated the differential phenotypic response under stress for yield, expressed as percent yield loss under irrigation vs drought (%YdL_D) and high vs low phosphorus (%YdL_LP). They were calculated: %YdL_LP = ((BLUP_higher P – BLUP_lower P) / BLUP higher P) * 100 (combining the trials Dar98, Pop99, Qui03b); and %YdL_D = ((BLUP irrigated – BLUP_drought) / BLUP irrigated) * 100 (Pal00 and Pal02 trials). For these BLUPs only data sets were used where stress vs non-stress conditions were evaluated in the same season. The same formula was applied for the calculation of percent loss in 100SdW, DF and DH.

### DNA, markers and QTL analysis

DNA was extracted from RILs and parents according to the method described before [43]. DNA quality was evaluated on 0.8% agarose gels followed by quantification on a Hoefer DyNA fluorometer (DNA Quant^™^ 200). The genetic map of the B x G population was constructed from 339 markers, from which 115 markers correspond to previous analyses [32] and we added 53 AFLP, 2 RAPD, 42 SSRs [44–48]. PCR amplification conditions were described before [44,49,50] and PCRs were conducted in 96-well plates using a PTC-100 thermal cycler (MJ Research, Watertown, USA). We also added 127 SNPs markers genotyped at LGC Genomics (www.lgcgenomics.com, Hoddesdon, UK) with KASP genotyping technology. These markers had been established by the Generation Challenge Program (https://www.generationcp.org/), based on the BARKBean chip [30,51].

The genetic map was constructed using MapDisto v. 1.7.5 (http://mapdisto.free.fr/), using Kosambi mapping function, with a minimum LOD score of 3 and maximum recombination fraction of 0.35; SARF and seriation as criteria for ordering and ripple. All genetic mapping was confirmed with the command for ‘‘best order” in MapDisto. Linkage groups were named based on the chromosome information of *Phaseolus vulgaris* genome v2.1 https://phytozome.jgi.doe.gov/pz/portal.html (consulted July 2018). QTL analysis was performed through QTL IciMapping software V4.0 based on the inclusive composite interval mapping of additive (ICIM-Add) [52]. The mapping parameters of each step for ICIM-ADD were set at 1.0 cM. LOD threshold value of 2.96 was applied for all traits, determined through completing 1000 permutations with the Type I error level set at α = 0.05 for each mapping method.

## Results

### Phenotypic data from four locations with abiotic stress treatments

**Fig 1:**
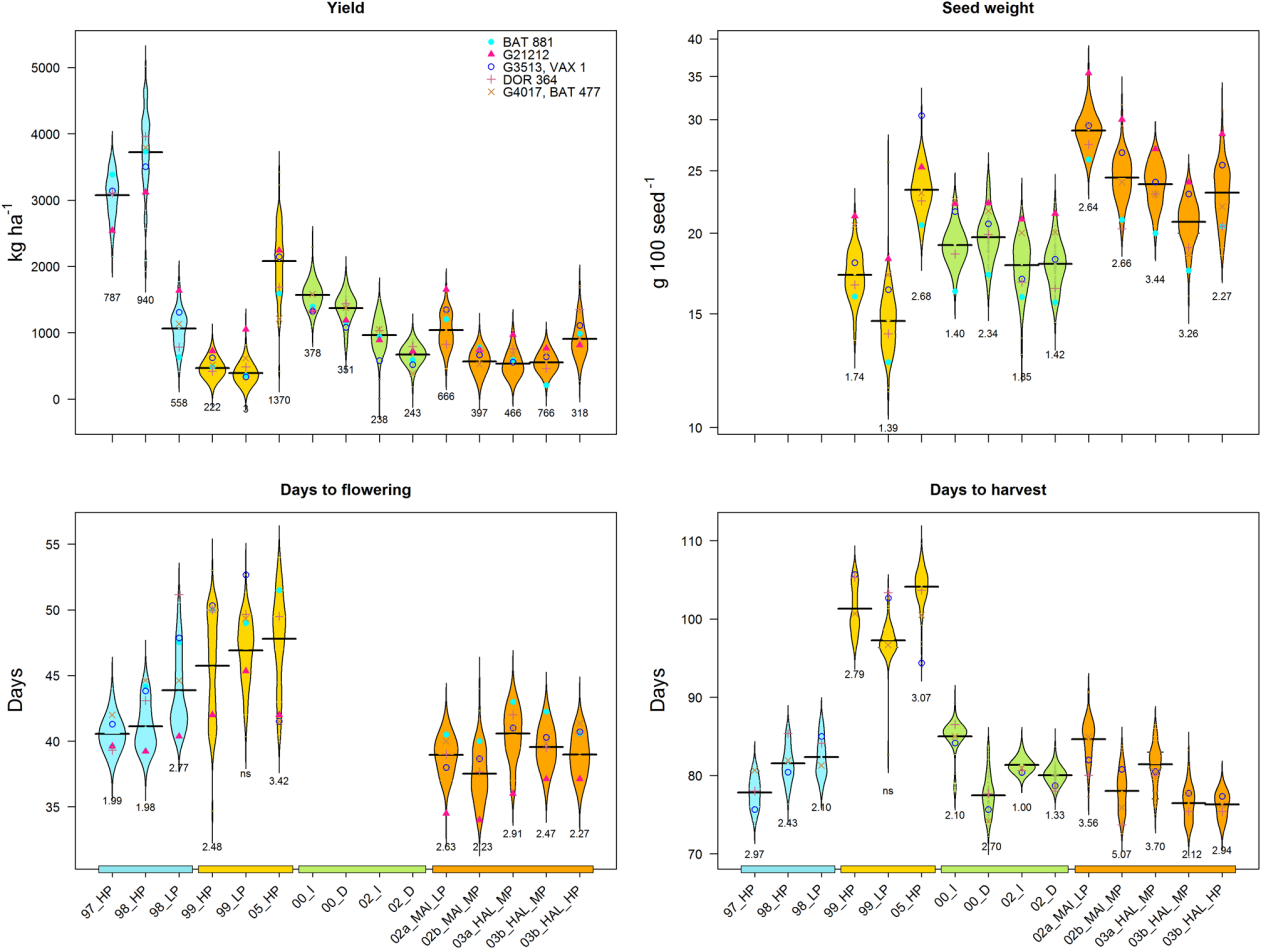
Phenotypic data of the BAT 881 x G21212 population, collected in 15 trials over four locations for the most frequently evaluated traits. On the x axis the trials appear in their respective year and treatments. Localities represented in colors; blue for Darién, yellow for Popayán, green for Palmira and orange for Quilichao. Treatments HP, MP and LP: high, moderate and low phosphorus, I: Irrigation, D: drought, HAl and MAl: High and moderate aluminum. Trait values of controls are marked with indicated symbols. G3513 and G4017 were only used at the localities of Darién, Popayán, Palmira, VAX1 and BAT 477 in Quilichao. LSD is noted below each plot.

The BAT 881 x G21212 RIL population was evaluated at four locations, totaling 15 trials with independent location/treatment combinations. Two trials were grown under drought stress, four in low P stress, and another three in high Al stress environments. A total of 31 traits were evaluated, only yield and DH were evaluated in all 15 trials (Fig 1 and Table 1), resulting in 225 data sets, including the averages for each locality for the major traits (Yd, 100SdW, DH, DF) and their overall averages. RILs presented continuous population distributions of traits by treatment (Fig 1 and S2 Fig). Transgressive segregation was also evident in B x G lines with lower or higher values compared to parents.

**Table 1:** Phenotypic evaluations of the BAT 881 x G21212 population in 15 different trials at four locations in Colombia.

Locations	Darien			Popayan			Palmira				Quilichao				
Year / Environment	97 HP	98 HP	98 LP	99 HP	99 LP	05 HP	00 D	00 I	02 D	02 I	02 MAl LP	02 MAl MP	03a HAl MP	03b HAl MP	03b HAl HP
**Yield components**															
Yd, Yield	3077	3737	1069	465	392	2098	1372	1579	671	975	1045	561	527	552	902
100SdW, Seed weight				17.2	14.6	23.3	19.7	19.1	17.9	17.9	28.9	24.4	23.9	20.9	23.2
PNo, Pod number						274			174	291		149	117	101	146
SdNo, Seed number						1198			663	1115		570	474	413	654
**Plant vigor**															
CB, Canopy biomass						2792	3656	3305	1912	3305	2188	1561	1105	813	1360
LAI, Leaf area index						1.809			1.740	2.350	1.955	1.273	0.936	0.950	1.477
LB, Leaf biomass						645	828	689	727	1015	687	448	329	320	470
SBMP, Stem biomass						715	897	673	518	840	634	248	282	267	450
PBMP, Pod biomass						1433	1932	1942	668	1449	866	1313	493	226	440
**Dry matter redistribution**															
HI, Harvest index						91.3			56.8	49.5		93.6	92.3	81.9	90.4
PHI, Pod harvest index						76.3			75.7	73.8		77.2	77.9	78.6	79.9
PPI, Pod partitioning index						119.2			75.0	66.5		120.7	118.2	104.1	113.0
TNC_Sh, Total non-structural carbohydrates in shoot									110	147	342	138	203	103	127
TNC_Sd, TNC in seed									276	266		393	376	383	367
**Phenological traits**															
DF, Days to flowering	40.6	41.0	43.7	45.6	46.8	47.9					38.9	37.5	40.5	39.5	38.9
DH, Days to harvest	77.9	81.6	82.4	101.3	96.7	104.2	77.6	85.0	80.1	81.4	84.8	78.0	81.5	76.5	76.3
**Mineral nutrients**															
ShN, Shoot nitrogen									2.78	2.66	2.45	3.57	2.92	3.54	3.27
SdN, Seed nitrogen							4.04	3.60	3.68	3.64		3.47	3.31		
NUE_Sh, N use efficiency in shoot									36.8	38.4	42.8	28.5	35.0	28.5	31.0
NUE_Sd, NUE in seed									11.7	10.2	18.3	15.6	16.9	17.8	18.4
ShP, Shoot P									0.304	0.345	0.231	0.281	0.298	0.282	0.314
SdP, Seed P							0.416	0.590	0.487	0.645		0.310	0.389	0.393	0.431
PUE_Sh, P use efficiency in shoot									338	296	443	361	344	361	324
PUE_Sd, P use efficiency in seed									105	78	190	196	167	228	193
**Differential stress responses**															
%YdL_D, Yield loss under drought								19.74							
%YdL_LP, Yield loss under low P								50.87							
%100SdWL_D, Seed weight reduction under drought								6.67							
%100SdWL_LP, Seed weight reduction under low P								8.44							
%DHL_D, Days to harvest reduction under drought								5.29							
%DHL_LP, Days to harvest reduction under low P								-0.21							
%DFL_LP, Days to flowering reduction under low P								-3.97							

Phenotypic means are shown, more details in S2 Fig. HP, MP, LP represent high, moderate or low P conditions. D: drought, I: irrigated. MAl and HAl: moderate and high Al.

### Drought and low P stress

Drought stress and non-stress treatments were compared in two seasons at Palmira (Pal) location. Drought reduced yield by 13% and 31%, respectively (Fig 1 and Table 1). Lowest precipitation among drought trials during pod filling stage was recorded in 2002 (88 mm vs 147 mm, S1 Fig), leading to stronger yield reduction and lower seed weight (Fig 1). Similarly, yield components PNo and SdNo were diminished in 2002, by 40% by and 41%, respectively, whereas seed size was not affected (S2 Fig). Biomass traits were significantly reduced by drought in the Pal 2002 trials (CB = 42%, LAI = 26% LB = 28%, SBMP = 38%, PBMP = 54%). In contrast, in Pal 2000 where the drought was less intense and more rainfall coincided with the pod filling phase no major reductions in biomass were observed. Dry matter redistribution trait values were elevated under drought, suggesting better allocation of dry matter resources to seed under stress (Table 1, S2 Fig). The lack of this data in the Palmira 2000 trial did not allow us to make a comparison between these traits between the two years. In most trials shoot traits LAI and LB showed positive yield correlations, however, in drought conditions negative yield correlations were observed (S2 Table). SdN was higher under drought conditions, whereas SdP was reduced.

Effects of low P stress were evaluated comparing stress and non-stress trials at three locations. Yield reductions were more severe in Darién (Dar) where low P reduced yields by 71% (Fig 1, S2 Fig, Table 1). In Popayán (Pop) yield was lowered by 16%, in a season probably affected by excessive rainfall (S1 Fig). In Pop99_HP exclusively positive yield correlations with DF and DH were found, in contrast, in Pop99_LP all correlations were negative (S2 Table). This condition-effect was not observed in Dar or Quilichao (Qui). In trials at Qui yields under moderate P levels (MP) were reduced by 39% compared to high P (HP) conditions. Effects of reduced P on yield components, biomass and dry matter redistribution traits were only evaluated in Qui. All were found to be diminished in moderate P conditions, including all dry matter redistribution traits which was unexpected. Even though shoot biomass production was reduced, lower values of HI, PHI and PPI indicate that seed formation and filling were even more compromised. SdP was reduced by 9% under low P conditions. Qui 2002 trials under moderate Al stress did not show the same tendencies as the trial in MP conditions out-yielded the HP treatment, however, these were sown in different seasons, where climatic differences likely having a larger effect on traits than soil P levels. In addition, good performance of Qui02_MAl_LP trial may be due to higher organic matter content in the soil (S1 Table).

**Fig 2:**
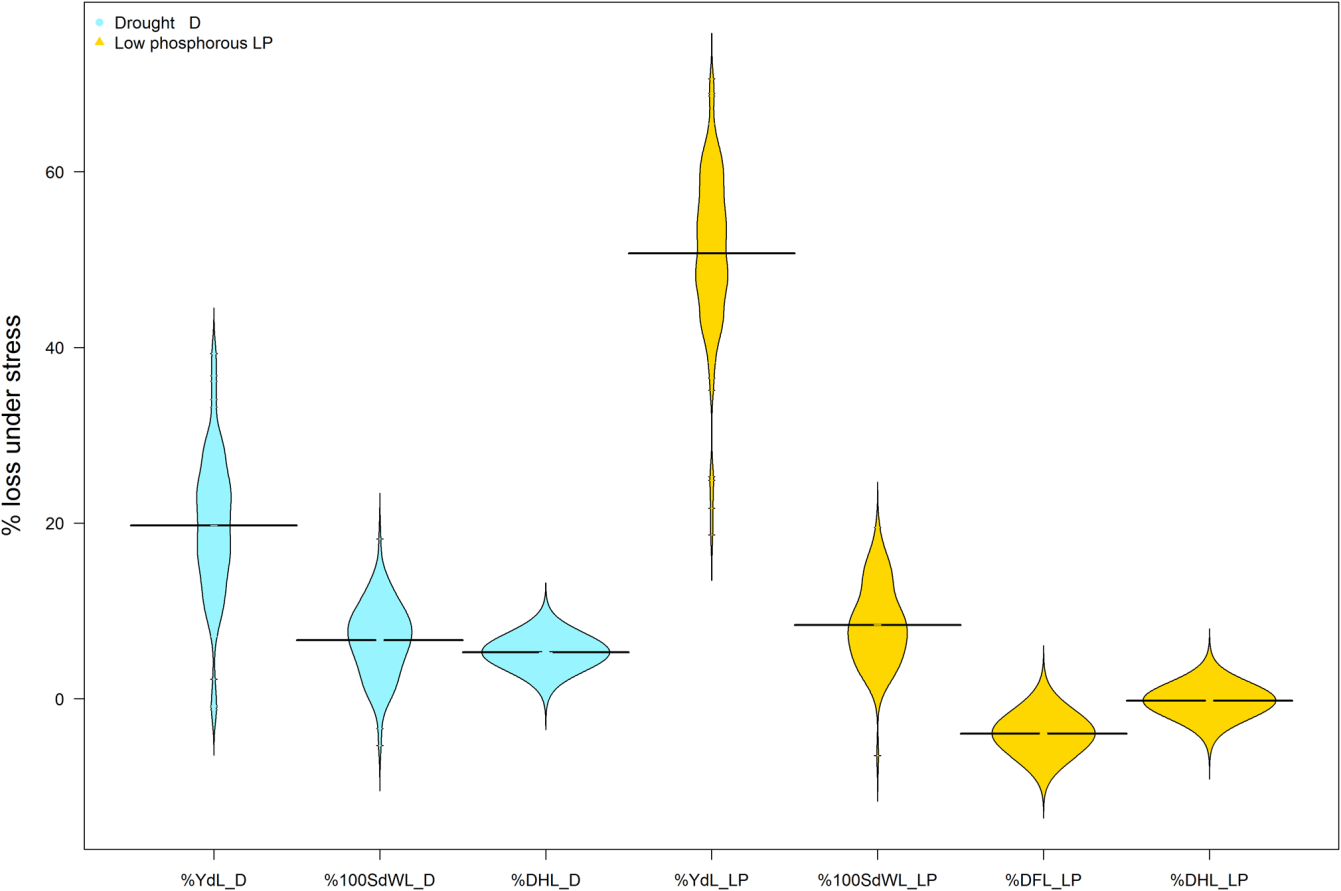
Frequency distributions of differential phenotypic responses under drought and low P conditions, expressed as % loss under stress for four major traits. BLUPs were used to join all available stress and non-stress data sets where stress and non-stress conditions had been evaluated in the same season. % loss under stress represented in colors; blue for drought (D) and yellow for low P (LP).

The differential phenotypic expression under stress was evaluated for the four major traits (Fig 2). Yield was reduced more under LP than under drought across these experiments. Most significant correlations between yield loss under stress (%YdL_D and %YdL_LP) and yield were as expected negative correlations in the respective stress trials (drought or LP, S2 Table). Furthermore, several positive significant yield correlations were found, so lines with high yields are more likely to see yields reduced under stress. Negative correlations were observed with pod and seed number as well as pod biomass, suggesting that these yield component traits aid stress tolerance. Positive correlations with maturity traits indicate that late maturing lines loose more yield under stress. Maturity traits saw more reductions in drought trials, under low P DF values were even extended. Accordingly, many more significant trait correlations were found for %DHL_D compared to %DHL_LP, generally contrasting the DH correlations.

Qui 2002 and Qui 2003 trials were planted on soils that were evaluated to have moderate and high Al levels, respectively. No significant effect attributed to differences in soil Al on yield and other traits could be detected across seasons.

**Fig 3:**
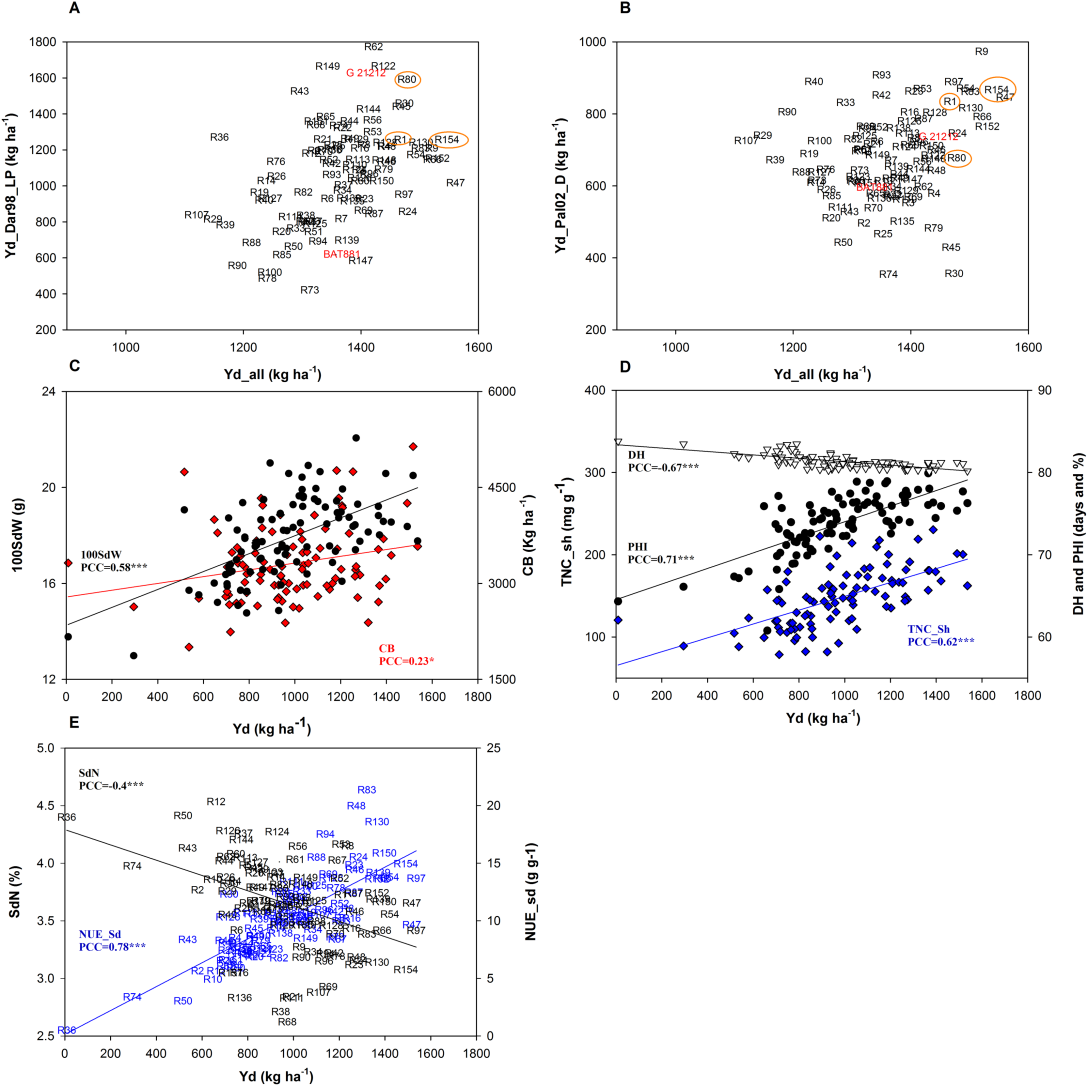
Phenotypic correlations of yield and related traits in selected trials. **A**: Correlation of yield under most severe low P stress and overall mean yield. **B**: Correlation of yield under most severe drought stress and overall mean yield. Best lines combining good stress performance and cross-location yield stability depicted orange; C-E, the most representative trial data set Pal02_I is used to show general tendencies. Estimated Pearson correlation coefficient (PCC) for each pair of traits. **C**: Correlation of 100SdW (black color) and CB (red color) with yield; **D**: Correlation of TNC_sh (blue color), DH (white) and PHI (black) with yield and **E**: Correlation of NUE_Sd (blue color) and SdN (black color) with Yd.

Superior RIL lines that performed well under stress and across trials were identified. Yields under most severe drought and low P stress trials were compared to average yields across all 15 trials (Fig 3A and 3B). High correlations indicate that most stress tolerant lines are among the most productive in all trials, displaying high general yield stability. This means that selection of best lines under stress conditions are also generally well performing under optimal conditions and vice versa. RILs R9, 47, 154, are the best lines to combine drought and cross-location yield stability, and another seven lines are excellent for both. For low P tolerance the correlation was also positive, but less clear as the top 5% of lines for each condition do not overlap. The best lines that combine cross-location yield stability and low P tolerance are R30, 45, 80, and the parental line G21212, which is excellent in low P. Taken together, drought and low P stress treatments had significant effects on productivity, yield components and biomass, however, variability between trials due to climatic and other conditions was also large. RILs 1, 80, and 154 can be useful as parents to develop multiple stress resistant genotypes.

Among the parental lines, G21212 parent showed higher yield and earliness than BAT 881 under LP stress in Pop and Qui, drought and combinations of high Al and moderate P (Fig 1). BAT 881 on the other hand, was superior under favorable conditions underlining the contrasting behavior of the parental lines. The additional controls G 3513 and G 4017 used in the Dar and Pop localities were similar in yield, their performance being generally intermediate between the two parents under low P. G 3513 performs poorly in Pal. The additional checks DOR 364, BAT 477 and VAX 1 selected for contrasting aluminum stress response are mostly inferior to G21212.

### Effect of locations on traits

**Fig 4:**
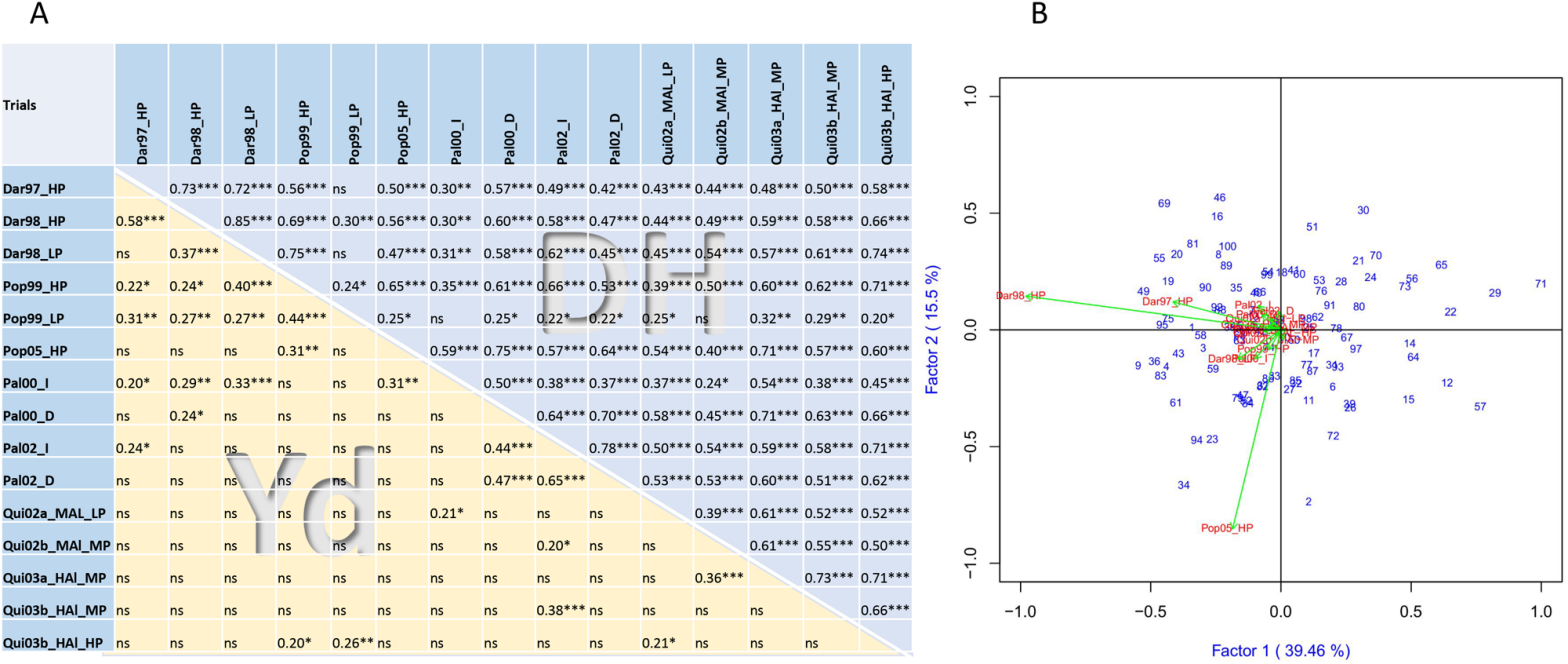
Phenotypic correlations and clustering between trials. A, phenotypic correlations between trials for days to harvest (upper right) and grain yield (lower left). Significance levels * p < 0.05, ** p < 0.01, *** p < 0.001. B, site regression analysis, showing clustering of trials based on yield data. RIL lines are noted with increasing performance in the direction of a specific trial vector.

Comparisons of phenotypic data sets showed strong correlations among trials. DH showed significant positive correlations between nearly all data sets (Fig 4A), demonstrating that the data set is useful for comparative analysis and variable response is highly stable. Yield correlations between locations were expectedly lower, all significant correlations were expectedly positive (Fig 4A). Best correlations were found between trials at Dar and Pop, which are at higher altitudes (1457–1730 masl, respectively) compared to lower altitude sites Pal and Qui (965–990 masl). Fewer significant yield correlations were observed between Pal and Qui locations, probably due to their contrasting soil fertility conditions.

Phenotypic data for major traits varied widely among trials (Fig 1). Highest yields were observed in Dar location under non-stress environments with average yields of 3.7 t/ha (Table 1). Pop was the most variable. Lower yields were observed in Pal and Qui. High altitude location Pop recorded most delayed flowering and maturity with highest DF and DH values. Highest seed sizes were observed at Qui location, a trait not evaluated at Dar.

Site regression analysis based on yield data (Fig 4B) resulted in Pop05_HP being the most distinct trial, Dar98_HP and Dar97_HP appear clustered to each other, different from all other data sets that appear in one large group. This result mainly mirrors average yield levels. Site regression did not generally result in distinct clusters for locations. Furthermore, no clustering of trials was observed that were subjected to the same conditions like drought, irrigation, high and low P. Variation between seasons due to climate or other factors seem to have larger effects on yield and other traits than locations or stress treatments.

### Yield, yield components and dry matter redistribution traits

Correlations between 224 trait data sets that were investigated are shown in S2 Table. Adjusted mean yield across all trials (Yd_all) was significantly positively correlated with all individual yield trials, underlining the general correlation among all experiments (Fig 4A). Location means for Dar and Pop highly correlated with trials from these sites, but not significantly with Pal and Qui trials, showing the differentness of the two higher altitude locations. For traits 100SdW and DF all trials and location means were highly correlated to each other. Taken together, this confirms that phenotypic data of all trials is comparable showing no drastic differences among trials.

Yield component traits PdNo and SdNo were, as expected, positively correlated with yield, as well as virtually all of 100SdW data sets (S2 Table). Regarding biomass traits, CB and PBMP show predominantly positive yield correlations. Within all 15 yield data sets, the Pal02_I trial had the highest number of correlations with other traits and also produced the highest number of QTLs. We interpret that as a high quality trial that can be used as a representative data set. Fig 3C shows positive yield correlations with CB and 100SdW in this most representative data set Pal02_I.

An evaluation of phenology traits revealed no connections between DF and yield in five trials in Dar and Pop, but significant negative yield correlations in all five Qui trials (not evaluated in Pal). Commitment to early initiation of reproductive development appears constitutively important in this population at lower altitude Qui location. This trend is equally clear in the DH trait, where all significant DH-Yd correlations were positive in Dar and Pop and negative in Pal (Fig 3D) and Qui. This shows different mechanisms for success, where extended seed fill is useful in cooler climates in high altitude locations, whereas early maturity is preferable in lower altitudes.

Dry matter redistribution and transport plays an important role in yield accumulation. PHI showed good yield correlations in Pal, Qui and Pop locations (not evaluated in Dar), reaching up to 0.71 in trial Pal02_I (Fig 3D). HI and PPI had positive yield correlations of lesser significance. Results underline utility of the PHI trait to select high yielding lines, holding up across seasons and locations and showing no particular preference for stress conditions. TNC is an interesting trait, with positive yield correlations if evaluated in shoots (Fig 3D, Table 2), but hardly in seed (S2 Table). Investigating further the relationship with biomass, reveals positive correlations with CB, but not with biomass of vegetative tissues, leaves and stem, where all significant correlations resulted negative (Table 2). Positive correlations were again observed with PBMP, hence the positive effect on CB (at mid pod fill) is solely due to pod mass. TNC_sh correlations were very negative with phenology traits DF/DH. Taken together, high TNC_sh levels lead to pod and seed formation and are not linked with vegetative biomass.

**Table 2:** Phenotypic correlations of total non-structural carbohydrates in shoot (TNC_sh) evaluated in 6 trials, with yield components and related traits.

Traits	TNC_sh	TNC_sh	TNC_sh	TNC_sh	TNC_sh	TNC_sh
Environment	Pal02_I	Qui02a_MAL_LP	Qui02b_MAl_MP	Qui03a_HAl_MP	Qui03b_HAl_MP	Qui03b_HAl_HP
**Yd_all**	0.22*	0.23*	ns	ns	0.21*	0.22*
**Yd_Pal**	0.40***	0.35***	ns	0.22*	0.33***	0.34***
**Yd_Qui**	ns	ns	ns	0.20*	ns	0.35***
**CB_Pop05_HP**	ns	0.33***	ns	0.25*	0.22*	ns
**CB_Pal00_I**	ns	0.25*	ns	ns	ns	ns
**CB_Pal02_D**	ns	ns	0.26**	ns	ns	ns
**CB_Qui03a_HAl_MP**	ns	ns	ns	0.26**	ns	0.25*
**CB_Qui03b_HAl_MP**	ns	ns	ns	ns	0.33***	0.33***
**CB_Qui03b_HAl_HP**	0.29**	ns	ns	0.37***	0.22*	0.39***
**LAI_Pal02_D**	-0.33***	ns	ns	-0.36***	-0.21*	-0.46***
**LAI_Qui02a_MAL_LP**	ns	ns	ns	ns	-0.2**	ns
**LAI_Qui02b_MAl_MP**	-0.20*	ns	ns	ns	ns	ns
**LB_Pal00_I**	ns	ns	ns	-0.2**	-0.35***	-0.23*
**LB_Pal00_D**	-0.2**	-0.25*	ns	-0.35***	-0.39***	-0.37***
**LB_Pal02_I**	-0.35***	-0.3**	-0.21*	-0.25*	-0.3**	-0.22*
**LB_Pal02_D**	-0.3**	-0.20*	ns	-0.37***	-0.24*	-0.44***
**SBMP_Pal00_I**	ns	ns	ns	ns	-0.38***	-0.2**
**SBMP_Pal00_D**	ns	ns	ns	ns	-0.35***	-0.3**
**SBMP_Pal02_I**	-0.24*	-0.33***	ns	-0.2**	-0.34***	-0.24*
**SBMP_Pal02_D**	-0.23*	-0.22*	ns	-0.39***	-0.2**	-0.45***
**SBMP_Qui02a_MAL_LP**	ns	ns	ns	ns	-0.21*	ns
**PBMP_Pop05_HP**	ns	0.39***	0.28**	0.31**	0.37***	0.28**
**PBMP_Pal00_I**	0.27**	0.34***	ns	ns	ns	ns
**PBMP_Pal00_D**	0.36***	0.20*	ns	0.24*	ns	ns
**PBMP_Pal02_I**	0.51***	0.34***	0.27**	0.38***	0.44***	0.45***
**PBMP_Pal02_D**	0.39***	0.31**	0.36***	0.41***	0.44***	0.37***
**PBMP_Qui02a_MAL_LP**	ns	ns	ns	0.22*	ns	0.30**
**PBMP_Qui02b_MAl_MP**	ns	ns	ns	ns	ns	0.21*
**PBMP_Qui03a_HAl_MP**	ns	ns	ns	0.40***	0.32**	0.38***
**PBMP_Qui03b_HAl_MP**	0.25*	ns	ns	0.26**	0.53***	0.50***
**PBMP_Qui03b_HAl_HP**	0.45***	0.23*	0.23*	0.55***	0.51***	0.60***
**DF_all**	-0.23*	-0.21*	ns	-0.41***	-0.58***	-0.44***
**DF_Dar**	-0.24*	-0.19*	ns	-0.39***	-0.57***	-0.45***
**DF_Pop**	ns	ns	ns	-0.33***	-0.50***	-0.34***
**DF_Qui**	-0.2**	-0.22*	ns	-0.43***	-0.56***	-0.45***
**DH_all**	-0.44***	-0.2**	-0.2**	-0.52***	-0.58***	-0.54***
**DH_Dar**	-0.37***	-0.25*	-0.2**	-0.52***	-0.60***	-0.54***
**DH_Pop**	-0.37***	-0.21*	-0.22*	-0.43***	-0.42***	-0.38***
**DH_Pal**	-0.40***	-0.2**	-0.22*	-0.37***	-0.45***	-0.45***
**DH_Qui**	-0.41***	-0.2**	-0.2**	-0.47***	-0.55***	-0.51***

Extract of complete correlation table in S2 Table, rows without significant correlations are not shown. Positive correlations in blue, negative in orange, correlations within one trial are indicated by a red box. Significance of correlation indicated as * p < 0.05, ** p < 0.001, *** p < 0.0001, ns = non significant. For full trait names, see Table 1.

All significant SdN—Yd correlations are negative within trials where both traits were scored (Fig 3E), SdP mainly follows the same tendency, but has one positive yield correlation in Qui. The negative relationship is likely a dilution effect from carbohydrate formation which has a higher variability due to genetic and environmental factors. Interestingly, also ShN and ShP showed only negative correlations with yield, and similarly yield components and TNC_sh. These traits were only measured in Pal and Qui locations. Furthermore, ShN evaluations were strongly positively correlated with nearly all DF and DH data sets, suggesting a link between late maturity and ShN. Hence higher protein/ammonia content of leaves may cause (or be caused by) increased vegetative development and later maturity, which has the aforementioned negative effect on grain productivity in these locations. Surprisingly this was not found for ShP, the only two significant correlations with DF/DH were negative, suggesting a different mechanism for P.

RILs R8, R53 and R67 combined good SdN with above average yield (Fig 3E), though, a solid negative correlation did not allow to identify lines that excel in both traits. Line R53 is above average on general, stress yield and SdN content and may be used for breeding to combine these traits. Taken together, combining high productivity and N content is not easy but to a certain extent possible. Yield components and dry matter redistribution traits PHI and TNC_sh are generally useful proxies for yield, whereas, effects of maturity and biomass traits are site and condition dependent.

### Identification of QTLs

**Fig 5:**
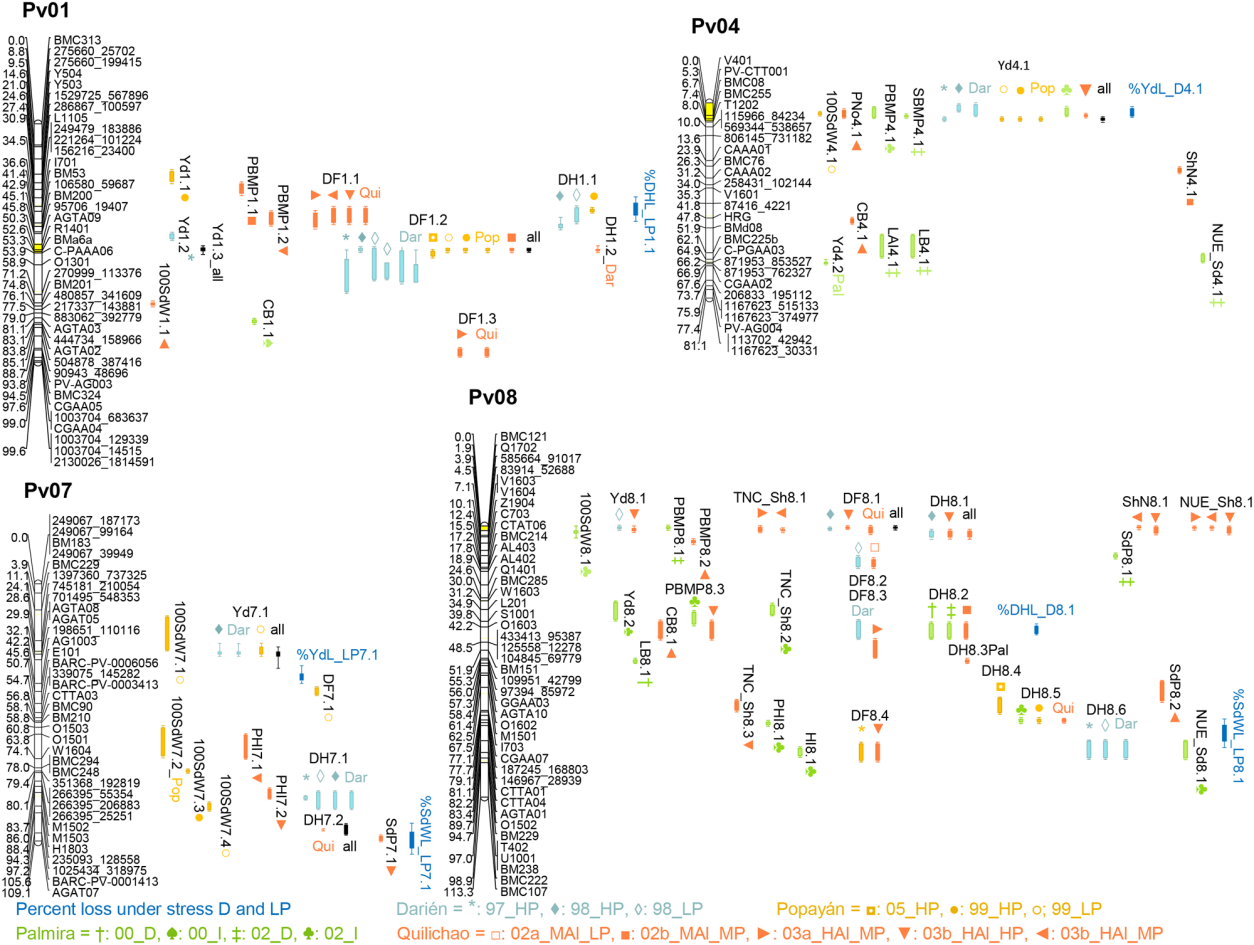
QTLs identified on chromosomes Pv01, Pv04, Pv07 and Pv08 of the BAT 881 × G21212 population. Colors depict the four locations (Darién in light blue, Popayán in yellow, Palmira in light green and Quilichao in orange), shapes indicate year and treatment (HP, MP and LP: high, moderate and low phosphorus, I: Irrigation, D: drought, HAl and MAl: High and moderate aluminum). Percent loss under stresses D and LP in light blue. Hotspots in yellow color.

An improved genetic map of the B x G population was generated that is suitable for QTL analysis. Genetic map B x G with 8 linkage groups, using a total of 115 markers covering 611 cM was previously reported [32]. We added AFLP (53), RAPD (2), microsatellite (42) and SNP (127) markers for a total of 339 markers, to improve map density and enable high precision QTL mapping in the B x G population (S3 Table). The total length of the genetic map is 1068 cM, which represents an increase of 457 cM over the previous map, with an average distance between markers of 3.15 cM. A total of 143 QTL were identified on all chromosomes for 25 of the 31 evaluated traits. 115 QTLs located in chromosomes Pv01, Pv04, Pv07 and Pv08 are shown in Fig 5 and listed in Table 3 (complete QTL list in S4 Table).

**Table 3:** Major QTLs identified in the BAT 881 x G21212 population. Complete list in S4 Table.

QTL name	Chr	Location environment	Pos (cM)	Left marker name	Right marker name	LOD	PVE (%)	Add	Source
**Yield components**									
Yd1.1	Pv01	Pop99_HP	22	Y503	1529725_567896	3.20	12.37	44.32	B
Yd1.2	Pv01	Dar97_HP	46	95706_19407	AGTA09	3.58	14.87	133.13	B
Yd1.3	Pv01	all	53	R1401	BMa6a	3.20	7.59	33.46	B
Yd4.1	Pv04	Dar98_HP	0	V401	PV-CTT001	3.80	15.66	-268.86	G
	Pv04	Dar	0	V401	PV-CTT001	4.78	19.16	-159.22	G
	Pv04	Pal02_I	4	V401	PV-CTT001	3.42	12.40	-92.76	G
	Pv04	Qui03b_HAl_HP	5	V401	PV-CTT001	2.91	14.34	-51.16	G
	Pv04	Dar97_HP	6	PV-CTT001	BMc08	4.29	19.14	-151.61	G
	Pv04	Pop99_LP	6	PV-CTT001	BMc08	5.09	20.47	-62.20	G
	Pv04	all	6	PV-CTT001	BMc08	9.87	29.29	-66.81	G
	Pv04	Pop99_HP	7	BMc08	BMc255	7.08	29.53	-69.08	G
	Pv04	Pop	7	BMc08	BMc255	3.69	18.25	-89.09	G
Yd4.2	Pv04	Pal	66	CGAA03	871953_853527	2.99	14.76	-54.02	G
Yd7.1	Pv07	Pop99_LP	26	745181_210054	701495_548353	5.64	23.04	-65.89	G
	Pv07	Dar98_HP	28	745181_210054	701495_548353	3.60	15.39	-247.88	G
	Pv07	Dar	28	745181_210054	701495_548353	3.84	15.45	-132.98	G
	Pv07	all	28	745181_210054	701495_548353	5.94	15.09	-47.51	G
Yd8.1	Pv08	Dar98_LP	0	BMc121	Q1702	5.49	24.96	-138.70	G
	Pv08	Qui03b_HAl_MP	1	BMc121	Q1702	3.14	16.13	114.62	B
Yd8.2	Pv08	Pal02_I	37	L201	S1001	4.63	16.55	104.84	B
100SdW4.1	Pv04	Pop99_LP	9	T1202	115966_84234	3.27	11.30	-0.45	G
**Percent yield loss under stress**									
%YdL_D4.1	Pv04	drought	15	806145_731182	CAAA01	3.72	16.00	-3.64	G
%YdL_LP7.1	Pv07	LP	43	AG1003	E101	2.50	12.53	6.24	B
%SdWL_LP7.1	Pv07	LP	93	H1803	235093_128558	2.54	10.08	1.42	B
%SdWL_LP8.1	Pv08	LP	90	O1502	BM229	3.84	14.61	-1.68	G
**Trait for vigor plant**									
PBMP4.1	Pv04	Pal02_I	5	V401	PV-CTT001	4.63	19.52	-179.90	G
SBMP4.1	Pv04	Pal02_D	5	V401	PV-CTT001	3.11	15.26	44.37	B
**Dry matter redistribution**									
HI8.1	Pv08	Pal02_I	95	BM229	T402	3.46	14.00	4.57	B
PHI7.1	Pv07	Qui03b_HAl_MP	69	O1501	W1604	3.90	23.21	0.70	B
PHI8.1	Pv08	Pal02_I	82	CTTA01	CTTA04	6.08	27.51	1.85	B
PHI7.2	Pv07	Qui03a_HAl_MP	89	H1803	235093_128558	3.60	16.44	0.91	B
TNC_Sh8.1	Pv08	Qui03a_HAl_MP	1	BMc121	Q1702	3.22	11.87	16.07	B
	Pv08	Qui03b_HAl_HP	1	BMc121	Q1702	3.39	13.45	10.97	B
TNC_Sh8.2	Pv08	Pal02_I	35	L201	S1001	3.74	17.92	14.57	B
TNC_Sh8.3	Pv08	Qui03b_HAl_MP	77	I703	CGAA07	4.29	17.72	17.06	B
**Phenological trait**									
DF1.1	Pv01	Qui03a_HAl_MP	36	156216_23400	I701	9.94	33.41	1.13	B
	Pv01	Qui03b_HAl_MP	36	156216_23400	I701	8.66	32.30	0.89	B
	Pv01	Qui	36	156216_23400	I701	13.16	37.09	0.89	B
	Pv01	Qui03b_HAl_HP	37	I701	BM53	6.60	24.49	0.78	B
DF1.2	Pv01	Pop05_HP	52	AGTA09	R1401	13.78	43.84	2.24	B
	Pv01	Dar98_HP	53	R1401	BMa6a	11.68	42.70	1.13	B
	Pv01	Dar98_LP	53	R1401	BMa6a	6.46	16.60	1.17	B
	Pv01	Pop99_LP	53	R1401	BMa6a	10.63	40.07	1.51	B
	Pv01	Pop99_HP	53	R1401	BMa6a	11.13	45.93	2.61	B
	Pv01	Qui02b_MAl_MP	53	R1401	BMa6a	13.27	47.85	1.43	B
	Pv01	Dar	53	R1401	BMa6a	6.57	16.67	0.73	B
	Pv01	Pop	53	R1401	BMa6a	13.43	52.60	2.15	B
	Pv01	all	53	R1401	BMa6a	13.87	46.87	1.24	B
	Pv01	Dar97_HP	58	CAAA06	O1301	5.54	23.15	0.61	B
	Pv01	Dar98_LP	59	O1301	270999_113376	5.56	13.36	1.09	B
	Pv01	Dar	59	O1301	270999_113376	5.43	12.78	0.66	B
DF1.3	Pv01	Qui	95	BMc324	CGAA05	4.52	10.59	0.48	B
	Pv01	Qui03a_HAl_MP	96	BMc324	CGAA05	3.69	10.76	0.64	B
DF8.1	Pv08	Dar98_HP	0	BMc121	Q1702	3.12	8.11	-0.50	G
	Pv08	Qui03b_HAl_MP	0	BMc121	Q1702	4.89	16.92	-0.65	G
	Pv08	all	0	BMc121	Q1702	3.40	8.45	-0.53	G
	Pv08	Qui	1	BMc121	Q1702	5.82	13.83	-0.55	G
DF8.2	Pv08	Dar98_LP	15	C703	CTAT06	4.14	9.56	-0.89	G
	Pv08	Qui02b_MAl_MP	15	C703	CTAT06	3.12	8.72	-0.61	G
DF8.3	Pv08	Dar	35	L201	S1001	4.39	10.06	-0.57	G
	Pv08	Qui03a_HAl_MP	40	S1001	O1603	4.61	12.90	-0.70	G
DF8.4	Pv08	Dar97_HP	94	O1502	BM229	4.18	16.66	-0.51	G
	Pv08	Qui03b_HAl_MP	94	O1502	BM229	4.80	16.46	-0.64	G
DH1.1	Pv01	Dar98_LP	35	156216_23400	I701	6.24	17.70	1.07	B
	Pv01	Pop99_HP	35	156216_23400	I701	4.19	16.60	1.22	B
	Pv01	Dar98_HP	41	I701	BM53	3.36	9.47	0.73	B
DH1.2	Pv01	Dar	53	R1401	BMa6a	4.58	12.83	0.77	B
DH8.1	Pv08	Dar98_HP	1	BMc121	Q1702	8.18	26.60	-1.23	G
	Pv08	Qui03b_HAl_MP	1	BMc121	Q1702	5.96	27.72	-1.20	G
	Pv08	all	2	Q1702	585664_91017	4.89	19.34	-0.72	G
DH8.2	Pv08	Pal00_D	36	L201	S1001	4.22	18.03	-1.06	G
	Pv08	Pal02_D	36	L201	S1001	6.25	27.49	-0.52	G
	Pv08	Qui02b_MAl_MP	36	L201	S1001	5.04	22.48	-1.24	G
DH8.3	Pv08	Pal	56	97394_85972	GGAA03	3.41	16.38	-0.51	G
DH8.4	Pv08	Pop05_HP	77	I703	CGAA07	4.28	20.13	-0.94	G
DH8.5	Pv08	Pal02_I	82	CTTA01	CTTA04	4.36	17.86	-0.34	G
	Pv08	Pop99_HP	82	CTTA01	CTTA04	4.94	19.96	-1.35	G
	Pv08	Qui	82	CTTA01	CTTA04	4.53	18.64	-0.81	G
DH8.6	Pv08	Dar97_HP	94	O1502	BM229	7.30	29.76	-1.12	G
	Pv08	Dar98_LP	94	O1502	BM229	9.88	32.23	-1.46	G
	Pv08	Dar	94	O1502	BM229	9.85	32.15	-1.23	G
**Percent DH loss under stress**									
%DHL_LP1.1	Pv01	LP	42	BM53	106580_59687	3.10	12.14	0.42	B
%DHL_D8.1	Pv08	drought	35	L201	S1001	3.60	17.24	0.54	B

Pos. cM: Genetic position in centimorgan; Physical pos: physical position in Mbp according to reference genome version 2.1; PVE (%): Phenotypic variation explained by the QTL; Add: Estimated additive effect of the marker; Sources: parents BAT 881 B or G21212 G that contribute the positive allele. For full trait names see Table 1.

A consistent yield QTL was identified at the start of chromosome 4 (Fig 5). QTL Yd4.1 was detected in 6 out of 15 trials in all four locations, including Al and low P stress treatments, and means across Dar, Pop, and all sites, indicating a very stable condition—independent QTL. The G21212 (G) derived allele explained up to 30% of phenotypic variability and boosted yield by an average of 112 kg/ha. Yd4.1 is co-localized with yield component QTLs for PBMP, PNo and 100SdW, positively correlated traits also supported by the G allele. Another yield QTL Yd7.1 was observed in two trials in Dar and Pop next to Dar location and across-locations yield means, explaining up to 23% also powered by the G allele. QTL for differential response under stress %YdL_D4.1 and %YdL_LP7.1 were discovered in these same regions. Also %DHL_LP1.1 and %DHL_D8.1 were found in similar regions as their corresponding trait, however, two QTL (%YdL_LP7.1 and %DHL_D8.1) were supported by the opposing allele. In some cases the positive allele that corresponds to higher values for the trait leads to more loss under stress, in the other cases the positive allele leads to more stress tolerance. %SdWL_LP8.1 is the only QTL that was solely identified for differential response under stress but not for the trait itself.

QTL hotspots for maturity were found on chromosome 1 and 8 (Fig 5). DF scored 31 QTL, the most of all traits even though not measured in all trials. On chromosome 1, 18 DF QTLs and 4 DH QTLs were detected, all supported by BAT 881 (B) parent; DF1.2 is strongest, explaining 12–52% of variation, data suggests three separate loci on this chromosome. On chromosome 8, 10 DF QTLs and 14 DH QTLs were detected, explaining up to 17% and 32% of variation, respectively. In this chromosome all positive alleles originate from G, data suggest ~5 independent loci. These two QTL hotspots for maturity cover the majority of the most significant genotype-phenotype associations.

For TNC_Sh several yield adjacent QTL were identified. TNC_sh6.1 was observed in two Qui trials, and co-localizes with Yd6.1 for Yd_all. The B allele explained 9–13% of variability for these traits. Furthermore, TNC_sh8.1 also noted twice in Qui, co-localizes with Yd8.2 from Qui, all supported by B allele. Nearby Yd8.1 found in Dar is based on the opposing G allele. Finally, TNC_sh8.2 co-localizes with Yd8.3 both from Pal02_I, again powered by the B allele. The four PHI QTL were not found to be condition-stable or co-localized to related relevant QTL. Taken together, TNC QTL co-localized with three yield QTLs, every time the B allele supports higher trait values.

## Discussion

### Bean productivity is affected by abiotic stress factors

This project produced a rich source of data, collected over 4 locations, 6 years, and three stress treatments. Nine out of fifteen trials had average grain yields lower than 1000 kg/ha, including most stress trials. This resembles typical farmer field yields in the tropics, hence, the data set is comparable and outcomes transferable to breeding efforts aimed at tropical smallholder farming systems.

Drought is the major abiotic stress affecting common bean and other legumes [53]. While approximately 67% of globally cultivated lands are affected by P deficits [54], low P tolerance in legumes has been mainly studied in common bean [53]. A deeper understanding of these traits is required to improve productivity under stress. In the B x G population drought and low P significantly reduced yields within evaluated location-season combinations, reductions of 13–31% at Palmira, 16% at Popayán, 71% at Darién and 39% at Quilichao were observed, in line with many field studies reviewed before in common bean, [18,23]. Drought stress caused yield reduction between 47 to 69% compared to the non-stress conditions [55], similarly, a reduction of 31% in the same conditions was reported [56]. For low P conditions compared to high P supply in same location reported yield reductions range between 50–60%. In other legumes, yield reductions due to stress have also been described in soybean, observing 36% yield differences under drought [57,58]. In drought trials, significant correlations with yield provided support for the important role that adaptation plays in selecting for drought resistance across environments, even though drought trials are inevitably noisier than non-stress trials. Yield correlations with vegetative biomass traits were found to be mostly negative, while correlations with overall biomass remained positive as previously reported [11,13]. Yield correlations with dry matter redistribution indices under drought were strong. Early maturity was strongly linked to increased productivity in trials in Qui and Pal. Next to drought avoidance and tolerance, drought escape of shorter duration varieties has been found to be an important trait in chickpea [59], cowpea [60] and lentil [61]. This indicates that focus on dry matter redistribution towards seed production has to be emphasized in water limiting conditions, as indicated before [62].

Similar to drought stress, low P conditions also reduced yields, yield components and biomass traits, in accordance with previous reports [63,64]. Interestingly dry matter redistribution trait values were reduced in the lower P treatment in Qui, the only low P trial in which these traits were evaluated. This contrasts observations under drought where the efficiency of dry matter distribution to grain was increased. In line with these findings, positive Yd-PHI correlations in low P along with reduced mean PHI values in low P were previously reported [64]. Retaining higher PHI under low P may represent a strategy for improved low P tolerance. QTL for differential trait response under stress were found mostly in the same regions as the corresponding traits. However, the alleles that lead to yield loss under stress are not always the same as those that lead to higher trait values indicating different mechanisms: Some alleles support high trait values under non-stress conditions and are thereby lined to higher losses under stress, and in other cases the allele supports the trait even more under stress. A significant effect of high Al stress could not be found as variability between seasons was high and contrasting treatments were only compared across seasons.

While drought and low P conditions significantly reduced yields, these impacts were overshadowed by cross-season and location effects that caused even more variation in yield and other traits. Late maturity is generally an advantage in higher altitudes, the opposite is observed in locations with lower altitude, overall, altitude was the major location effect observed. Soybean was found to have longer growth cycles but lower yields in higher altitudes [65] but in general few studies exist on this topic that deserves more attention.

Site regression analysis based on yield did not result in clear location clustering, unlike maturity traits that were found to be very location dependent. This suggests that even though yield correlations are more significant among locations in high or low altitudes, selection in all locations can be utilized to improve lines for all environments. Lines were identified that displayed good performance under strong stress and good cross-location yield stability, at least for the range of environments investigated here. This shows that improvement through breeding for these traits can be achieved and that selection under extreme stress does not carry a penalty for productivity under optimal conditions [3,26]. This is an important finding to plan selection in breeding programs, allowing identification of lines with good cross-location productivity, rather than requiring site specific breeding activities.

### Productivity dependence on yield components and dry matter redistribution

Yield showed expected solid correlations with yield component traits and biomass traits, in line with many previous reports [11,16,62,64]. Several groups reported positive yield correlations of 100SdW in stress and non-stress conditions [14,62,66], other reports showed positive yield correlations only in stress conditions [13,15,64], occasionally even negative in non-stress and positive under stress [67]. In this population 100SdW was always positively linked to yield, seed fill and seed size are required for yield and not replaced by a larger number of smaller seed. In this work no major QTL hotspots for 100SdW appeared which are often found in such projects [50,64,68], indicating that there may be no major gene variability for 100SdW in this population.

Previous reports on yield correlations with maturity traits have been diverging. Several negative yield correlations were reported [14,64,69], suggesting that earliness aids productivity in stress as well as non-stress conditions. Other reports observed significant negative correlations only in stress conditions [11], or even contrary results between environments, with positive correlations under non-stress conditions [13,70], up to strong positive yield correlations of maturity in all conditions [62]. In this work in Pal and Qui locations DF/DH were always negatively correlated with yield. On the other hand, enhanced vegetative development contributed to increased productivity at Pop and Dar sites, hence this is rather an environment dependent effect and not due to the specific genetic background. Multi-location comparisons of effects of maturity as shown in this data set have not been published before with several major abiotic stress factors, this helps to put previous reports in perspective. Diverging correlations between yield and maturity are rather due to differences between evaluated environments than evaluated germplasm.

PHI continues to be a good proxy for yield, displaying positive yield correlations in all evaluated conditions. This confirms that effective seed fill is a useful selection criteria. Some previous publications reported correlations of PHI with seed yield in all conditions [13,14,16,62,66], while in others PHI only had significant yield correlations under stress conditions [15,64,67]. In this work the effect does not appear to be stress specific. It also appears to be useful in cooler climates in higher-altitudes.

TNC_sh proved to be an interesting trait that is linked to yield formation. TNC levels were reported to be positively correlated with starch in storage tissue of legume roots [71]. In soybean studies drought was found to reduce pod number and yield accompanied by an increase of %TNC in pods, showing that lower sink strength increases %TNC [72]. In this study positive correlations with yield, total and pod biomass, but not with vegetative plant tissue suggest two possible source-sink relationships: the active vegetative growth tissues are competing for TNCs thereby reducing yield, or the variability in TNC levels lies in the variability in photosynthetic activity of source tissues (leaves) to provide enough energy for seed production. Bush beans usually cease vegetative development at some point, which reduces sink (young) tissues, thereby increasing TNC levels and TNC uptake by reproductive sink tissues. Increased TNCs through photosynthetic activity should correlate with both increased yield and leaf and stem biomass. As the latter is not observed, the first relationship is more likely. Vegetative tissues dilute and use sugar and starch not fully committing to their source role at crucial stages and thereby deprive pods and seed from energy for generating yield. TNC_sh represents a powerful selection trait, but with current technology may be too cumbersome to phenotype to be utilized in large scale breeding.

In any environment biomass is still a prerequisite for yield, a large, strong plant is needed as a basis, hence, the resource allocation into vegetative and reproductive development needs to be balanced. Plants need to grow large quickly, and then fully commit to reproductive development without unnecessary sinks to fill seed before senescence sets in. In cool conditions at Dar and Pop sites the process is not so confined in time, and an extended seed fill phase with larger DH, not DF, aids productivity.

### Major QTL affect yield and maturity

G21212 was earlier maturing than BAT 881, observed in 25 of 26 DF and DH evaluations in the 15 trials (Fig 1). QTL analysis reveals the genetic basis of this observation, which is likely the maturity QTL hotspot in chromosome 1, with 22 identified maturity QTLs over all data sets. DF1.2^BG^ is the strongest QTL found in this study, the allele of BAT 881 increased flowering time on average by 1.2 days. QTL for DF on chromosome 1 were previously reported showing location stable DF QTL on the start and end of chromosome 1, next to a days to harvest maturity QTL [68,73]. In the same region of DF1.2, near marker BM201 the *fin* locus was reported, that determines the determinate growth habit. The *fin* locus has been related to changes in architecture and growth period that may help the plant to tolerate stresses [74], however, both parental lines have an indeterminate growth habit. Data in this study suggests several loci (4–5) (Table 3) with similar effects and source, for both maturity hotspots on chromosome 1 and 8. Genetically this is not very credible, the authors assume that there is only one locus per chromosome, QTL mapping software may not pick up the precise location range. In summary, one of the clearest physiological differences observed between parental lines can be explained with the most significant QTL.

The most exciting discovery of the QTL analysis is the identification of a fairly stable yield QTL. Yd4.1^BG^ was found in all four locations, high P, low P, as well as high Al and irrigated conditions. G21212 is higher yielding in most trials, and its allele at Yd4.1^BG^ explains up to 30% of variation (average of 112 kg/ha yield boost). Stable yield QTL are rare to find. A yield QTL was reported on Pv02 in two out of four trials only in non-stress conditions [50], and similarly a QTL on Pv03 was reported in two out of seven trials [66]. A condition stable QTL was published on Pv07 but observed in only two out of two trials [64]. An interesting seed yield QTL SY1.1^BR^ was discovered in five out of 12 trials over three locations, but not across stress treatments in any location [68]. The unusually strong and condition-independent yield QTL Yd4.1^BG^ found in this study is not clearly associated to another trait or a mechanism, unlike e.g. the reported QTL SY1.1^BR^ [68] which is a QTL for delayed flowering time. Other examples are the yield and flowering time/ growth habit QTL in tomato [75], or DRO1 QTL that modifies root architecture in rice [76]. The mechanism of action of Yd4.1^BG^ is unknown, but the absence of obvious physiological effects that may be undesirable for breeders suggests that it is easier to introgress into elite breeding material. Dry matter redistribution traits PHI and TNC_sh were found to correlate with yield in several trials. Three TNC_sh QTL co-localized with yield QTL, providing a mechanistic explanation for these QTL, however, the effects are not as strong and stable as Yd4.1^BG^ or the maturity hotspots. Direct molecular markers linked to yield have been sought after for several years with considerable investments, but their application in breeding is scarce in any crop and not reported in common bean. Yd4.1^BG^ represents the best candidate for use in MAS. Sequencing data from a panel will be used to identify G21212 specific SNPs tagging this locus aiming to employ it in diverse genetic backgrounds.

## Summary and conclusion

This comprehensive study produced a rich source of data from 15 field trials, quantifying significant yield losses under different abiotic stresses. The study of dry matter redistribution to grain proved to be important under all conditions, and PHI and TNC_sh allowed indirect selection of higher yielding lines. Early maturity aids productivity in lower altitudes, in contrast, in higher altitudes an extended growth season is generally of advantage. Selection under strong stress conditions identifies lines that are also performing well under non-stress conditions, an important information that can be applied in selection process. QTL analysis revealed a stable yield QTL, Yd4.1 that showed significant association in all locations, next to two strong maturity QTL hotspots. Yd4.1 is the best candidate for marker assisted selection (MAS).

Drought and low P are among the major limitations affecting the production of common bean and many other crops in the tropics. Understanding which traits are associated with productivity under expected increase of drought and extreme temperature conditions due to climate change can guide research and germplasm development to increase yields in stress and non-stress conditions. Data obtained in this study will help bean breeding efforts through information on germplasm and development of molecular tools.

## Supporting information

S1 Fig Maximum and minimum temperatures, rainfall distribution and pan evaporation during crop growing period at 2002–2003 Quilichao; 2000 and 2002 Palmira and 1999 and 2005 Popayán in Colombia. (TIF)

S2 Fig Frequency distributions of phenotypic traits evaluated in 15 trials over four locations for the BAT 881 × G21212 population. Blue circle and pink triangle indicate phenotypic values for BAT 881 × G21212, respectively. LSD shown under each violin plot.
(TIF)

S1 Table Soil conditions for 15 trials across the four locations Darién, Popayán, Palmira and Quilichao where the population BAT 881 x G21212 was evaluated. (DOCX)

S2 Table Pearson correlations between phenotypic trait data sets. Only significant correlations are shown, with significance levels of * = p < 0.05, ** = p < 0.01, *** = p < 0.001. Significant positive correlations depicted in blue, negative correlations in red. Correlations within the same trial data set are boxed in red.
(XLSX)

S3 Table Summary of molecular markers and mean distances between markers on individual chromosomes in the genetic linkage map of the BAT 881 x G21212 population. (DOCX)

S4 Table All QTLs identified in the BAT 881 x G21212 population evaluated at four locations in Colombia. Pos. cM: Genetic position in centimorgan; Physical pos: physical position in Mbp, according to reference genome version 2.1; PVE (%): Phenotypic variation explained by the QTL; Add: Estimated additive effect of the marker; Sources: parent BAT 881 B or G21212 G that contributes the positive allele. For full trait names, see Table 1.
(XLSX)
