# Supplementary figures and images for: QTL analyses for tolerance to abiotic stresses in a common bean (Phaseolus vulgaris L.) population
Source: PLoS One. 2018 Aug 29;13(8):e0202342. doi: 10.1371/journal.pone.0202342 (PMC6114847; doi:10.1371/journal.pone.0202342)

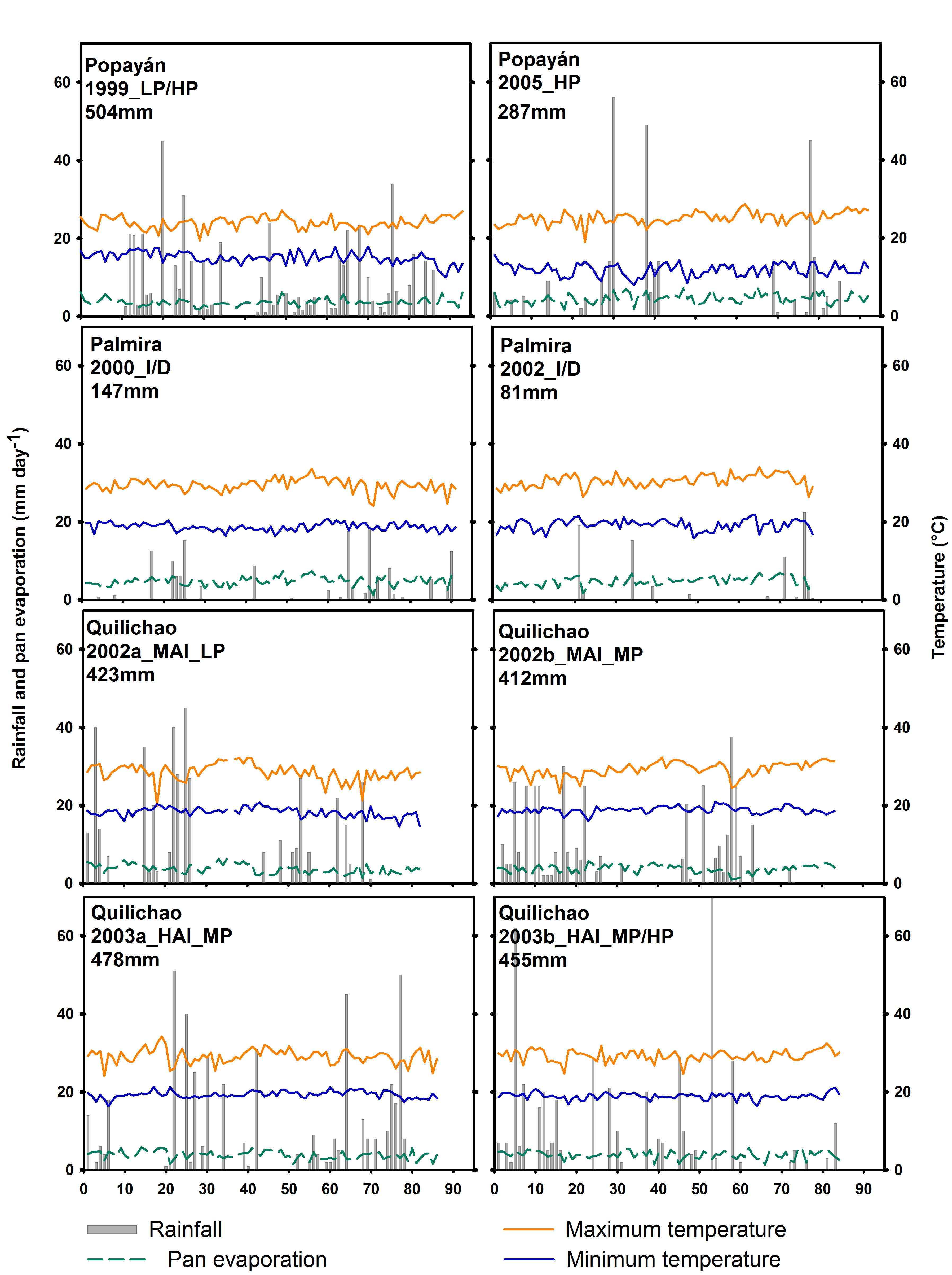

Supplement: S1 Fig — (TIF) [file pone.0202342.s001.tif]

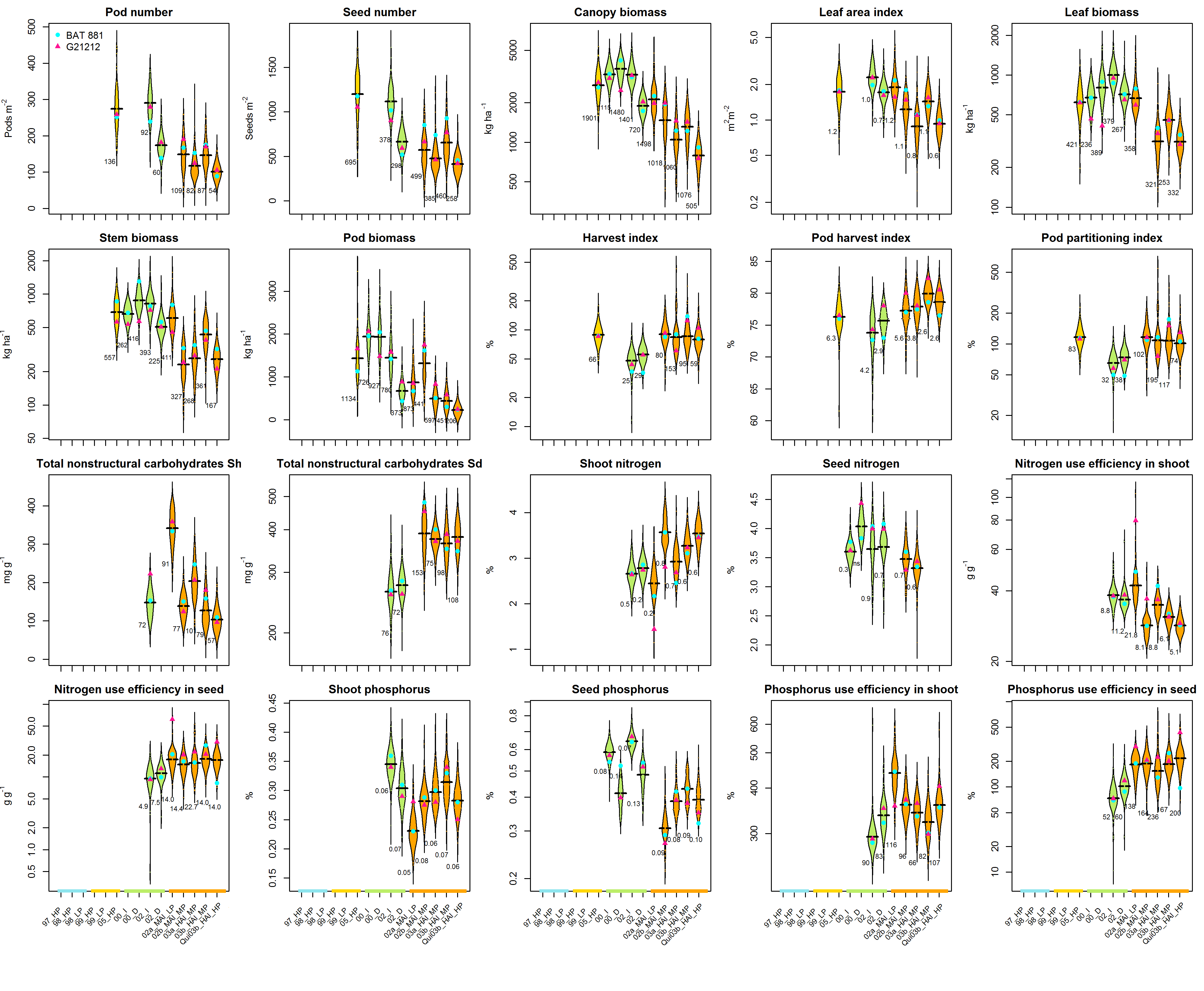

Supplement: S2 Fig — Blue circle and pink triangle indicate phenotypic values for BAT 881 × G21212, respectively. LSD shown under each violin plot. (TIF) [file pone.0202342.s002.tif]
