# Supplementary material for: QTL analyses for tolerance to abiotic stresses in a common bean (Phaseolus vulgaris L.) population
Source: PLoS One. 2018 Aug 29;13(8):e0202342. doi: 10.1371/journal.pone.0202342 (PMC6114847; doi:10.1371/journal.pone.0202342)
Supplement: S1 Table — (DOCX) [file pone.0202342.s003.docx]

**Suppl. Table 1. Soil conditions for 15 trials across the four locations Darién, Popayán, Palmira and Quilichao where the population BAT 881 x G21212 was evaluated.**

| **Trails** | **Al saturation (%)** | | **P (µg g-1)** | | **pH** | | **OM (%)** | |
| --- | --- | --- | --- | --- | --- | --- | --- | --- |
| Soil depth(cm) | 0-10 | 10-20 | 0-10 | 10-20 | 0-10 | 10-20 | 0-10 | 10-20 |
| ***Darién*** *(1457 masl, inceptisol)* | | | | | | | | |
| Dar97_HP | 8 |  | 6.70 |  | 5.26 |  | 6.65 |  |
| Dar98_HP | 8 |  | 6.70 |  | 5.26 |  | 6.65 |  |
| Dar98_LP | 9 | 8 | 1.20 | 0.80 | 5.37 | 5.26 | 10.45 | 10.49 |
| ***Popayán*** *(1730 masl, inceptisol)* | | | | | | | | |
| Pop99_HP |  |  | 4.50 | 4.50 | 6.35 | 6.33 | 14.28 | 13.69 |
| Pop99_LP |  |  | 7.80 | 7.50 | 6.11 | 5.99 | 17.87 |  |
| Pop05_HP |  |  | 8.35 |  |  |  |  |  |
| ***CIAT- Palmira*** (*965 masl, mollisol*) | | | | | | | | |
| Pal00_D |  |  | 69.30 | 58.40 | 7.60 | 7.56 | 5.76 | 6.33 |
| Pal00_I |  |  | 64.10 | 54.10 | 7.52 | 7.62 | 7.15 | 6.15 |
| Pal02_D |  |  | 73.80 | 71.60 | 7.74 | 7.73 | 4.71 | 4.52 |
| Pal02_I |  |  | 64.10 | 54.10 | 7.52 | 7.62 | 7.15 | 6.15 |
| ***Quilichao*** (*990 masl, oxisol*) | | | | | | | | |
| Qui02a_MAl_LP | 40 | 64 | 3.30 | 1.90 | 4.50 | 4.46 | 10.70 | 9.05 |
| Qui02b_MAl_MP | 43 | 45 | 17.10 | 6.90 | 4.43 | 4.46 | 5.63 | 5.24 |
| Qui03a_HAl_MP | *61* | *62* | 19.30 | 10.30 | 4.00 | 4.01 |  |  |
| Qui03b_HAl_MP | 64 | 67 | 13.20 | 5.50 | 4.22 | 4.16 | 6.10 | 5.41 |
| Qui03b_HAl_HP | 61 | 61 | 20.30 | 17.50 | 4.22 | 4.21 | 5.59 | 5.41 |

Trial descriptors contain location (Dar: Darién, Pop: Popayán, Pal: CIAT-Palmira, Qui: Quilichao), year, and treatments (HP, MH and LP: high, moderate and low phosphorus, I: Irrigation, D: drought, HAl and MAl: High and moderate aluminum). OM: organic matter
