# Supplementary material for: QTL analyses for tolerance to abiotic stresses in a common bean (Phaseolus vulgaris L.) population
Source: PLoS One. 2018 Aug 29;13(8):e0202342. doi: 10.1371/journal.pone.0202342 (PMC6114847; doi:10.1371/journal.pone.0202342)
Supplement: S3 Table — (DOCX) [file pone.0202342.s005.docx]

S3 Table. **Summary of molecular markers and mean distances between markers on individual chromosomes in the genetic linkage map of the BAT 881 x G21212 population.**

| **Chr.** | **AFLP** | **RAPD** | **SNP** | **SSR** | **Total markers** | **Map size cM** | **Mean distance between markers in cM** |
| --- | --- | --- | --- | --- | --- | --- | --- |
| **1** | 6 | 6 | 20 | 7 | 39 | 99.62 | 2.55 |
| **2** | 1 | 7 | 17 | 5 | 30 | 90.00 | 3.00 |
| **3** | 4 | 9 | 3 | 7 | 23 | 99.82 | 4.34 |
| ***4*** | *3* | *4* | *13* | *7* | *27* | 81.10 | *3.00* |
| **5** | 8 | 11 | 14 | 4 | 37 | 107.58 | 2.91 |
| **6** | 7 | 17 | 5 | 9 | 38 | 124.54 | 3.28 |
| **7** | 5 | 7 | 17 | 6 | 35 | 109.06 | 3.12 |
| **8** | 7 | 18 | 9 | 8 | 42 | 113.33 | 2.70 |
| **9** | 1 |  | 11 | 6 | 18 | 69.26 | 3.85 |
| **10** | 6 | 7 | 5 | 4 | 22 | 72.95 | 3.32 |
| **11** | 5 | 5 | 13 | 5 | 28 | 100.94 | 3.61 |
| **Total** | **53** | **91** | **127** | **68** | **339** | **1068.20** | **3.15** |
